# Pandora's Box

**DOI:** 10.1192/bji.2023.6

**Published:** 2023-05

## ‘Standing on the shoulders of giants’ but no leaps in the air

Research in all areas of science has increased enormously over recent years for a variety of reasons including pressure to publish for career purposes, technological progress and others. However, this ever-increasing productivity does not seem to be associated with any significant increase in innovation according to the authors of a recent survey. Based on theories of scientific and technological change, which view discovery and invention as endogenous processes, they claim that the vast accumulated scientific and technological knowledge should make it easier for major advances, quoting Newton who humbly attributed his major discoveries to ‘standing on the shoulders of giants’. They considered various explanations for this, including the increasing burden of knowledge requiring longer training to reach the point of being able to push the frontiers forward.

The authors tried to understand the reasons behind this relative decline by analysing data on 25 million papers (1945–2010) in the Web of Science and 3.9 million patents (1976–2010) and replicated their findings using four additional data sources – JSTOR, the American Physical Society Corpus, Microsoft Academic Graph and PubMed, encompassing 20 million papers, together with the CD index quantitative metric, which characterises how papers and patents change networks of citations in science and technology. They confirmed that both papers and patents are increasingly less likely to break with the past and push science in new directions. They found that this pattern holds across fields and is robust across multiple different citation and text-based metrics. Chu and Evans (2021) observed that scholars in fields where an abundance of papers are published have significant difficulties getting published, read and cited if their work does not contain references to already widely cited articles. They claim that this stagnation is to do with researchers’ tendency to cite the same papers, particularly those with higher numbers of citations. Gomez et al (2022) blame the lack of progress on the bias of the scientific communities in favour of highly active countries, while overlooking work from peripheral countries.

Drastic changes to our attitudes are obviously needed if we want to see major breakthroughs, particularly in psychiatry. According to the authors, progress can only be made if quantitative growth in scientific endeavours such as numbers of scientists, institutes and papers is accompanied by structures fostering ‘disruptive’ scholarship and focusing attention on novel ideas.

Park M, Leahey E, Funk RJ. Papers and patents are becoming less disruptive over time. Nature 2023; 613: 138–44.36600070 10.1038/s41586-022-05543-x

Chu JSG, Evans JA. Slowed canonical progress in large fields of science. Proc Natl Acad Sci USA 2021; 118(41): e20216361.10.1073/pnas.2021636118PMC852228134607941

Gomez CJ, Herman AC, Parigi P. Leading countries in global science increasingly receive more citations than other countries doing similar research. Nat Hum Behav 2022; 6: 919–29.35637294 10.1038/s41562-022-01351-5PMC9314251

## Hope on the horizon for future academics

Chris Woolston reports, in *Nature*, about the European Union initiative that proposes a new approach to funding and university appointments, moving away from metric measures such as impact factors and *h*-indices. It is suggested that universities, scientific academies, funding institutions and other relevant organisations around the world be offered the option to sign a document that commits them to changing the way they assess researchers for jobs, promotions and grants. The signatories would be expected to commit to moving away from standard metric assessments and use instead a system that rewards researchers for the quality of their work and their full contribution to science. The document is known as the *Agreement on Reforming Researcher Assessment.* This was endorsed by the European Commission, which proposed that the assessment criteria reward ethics and integrity, teamwork and a variety of outputs alongside research quality and impact. Over 160 European Universities and 190 other research organisations expressed support for the initiative. Encouraging as these numbers may seem, it should be considered that the European Union has 850 universities. In the UK, only the University of Glasgow and Loughborough University have considered taking part. Time will show whether other institutions will be prepared to move in this direction.

The European Union proposal does not dismiss metrics such as journal impact factor and *h*-index that measure research productivity, but the advice is not to misuse these. Interestingly, a Dutch University has abandoned the use of impact factors in hiring and promotion decisions. It also calls for an end to considering the ranking or reputation of a researcher's institution when making appointment decisions, delivering awards or granting funding.

Although created by European institutions, the initiative is not just a ‘European issue’ and it is hoped that institutions anywhere in the world will consider joining.

Woolston C. Grants and hiring: will impact factors and *h*-indices be scrapped? *Nature* [Epub ahead of print] 19 Sep 2022. Available from: 10.1038/d41586-022-02984-2.36123531

## Mind over matter, but how?

Doctors and more so psychiatrists are well aware of the important role of our mental state – in particular, stress – in the genesis and outcome of many physical conditions in addition to mental ones such as depression. There is a close relationship and a complex interaction between the nervous and immune systems, and ample evidence is available showing that psychological stress modulates immune function. However, the mechanics of this are still to be fully unravelled.

The recent Covid pandemic and the urgent need for effective vaccines revitalised research interest in viral infections and the body's immune response. In a recent paper, Poller and colleagues endeavoured to shed some light on the subject, focusing on the way stress networks in the brain link to peripheral leucocyte dynamics relevant to the fight against viral infection. Using optogenetics and chemogenetics, they carried out a series of experiments in mice. They first demonstrated that in the early period of acute stress, the number of circulating lymphocytes and monocytes is decreased while the number of neutrophils is increased. Searching for the source and the destination of the leucocyte migration, they found that acute stress mobilises neutrophils out of the bone marrow. By contrast, lymphocytes and monocytes are redistributed from lymphoid organs to the bone marrow.

In further experiments, they examined the role of brain mechanisms associated with stress, i.e. the HPA (hypothalamo–pituitary–adrenal) axis and the sympathetic nervous system, in controlling these peripheral white cell dynamics. They found that under acute stress conditions, the HPA axis activates a bone-marrow-based chemokine that controls the observed migration of lymphocytes and monocytes from lymphoid tissues and the blood to the bone marrow.

The authors’ data show that the combination of stress-induced neutrophilia and lymphopenia impairs the body's ability to fight viral infection. The acute stress-induced mobilisation of neutrophils in the blood circulation encourages their infiltration of peripheral organs, where they can participate in inflammation. Stress-induced reduction of lymphocytes in the blood circulation and lymph nodes decreased their ability to survey the presence of antigens, impairing the detection of and consequently the fight against viral infections.

The authors, recognising that further studies are needed to better understand the neuroimmune pathways, conclude that the physiological adaptation to acute stress not only elicits fight or flight behaviour but also modulates the body's response to infection. Their data show how specific neuron clusters in the brain relevant to fear cause massive changes in leukocyte distribution and function and demonstrate the significant negative effect stress can have on the body's ability to fight illness, at least as far as viral infection is concerned.

Poller WC, Downey J, Mooslechner, Khan N, Li L, Chan CT,  Brain motor and fear circuits regulate leukocytes during acute stress. Nature 2022; 607(7919): 578–84.35636458 10.1038/s41586-022-04890-zPMC9798885

## Do you know your own mind?

As humans, we are social beings and our behaviour can be influenced by our social networks. Researchers in Beijing, China, examined how our brains make decisions in this networked environment. They focused on three areas, all related to how we learn about an unknown environment from the decisions of one or more individuals observed performing the same task as ourselves. First, how the brain learns from peer connections. Second, when there is divergence in the behaviour of individuals in the same networked environment, how it evaluates the relative importance of each observation. Third, the extent to which such divergence relates to the relative position of an individual within the network. They built a model network structure of dependent learning, drawing insights from the reinforcement learning literature, neural computational studies of social and observational learning, and social network analysis of decision heuristics in imitation and social influence settings.

Participants were randomly allocated to different parts of the network. They played an online game, the objective of which was to adapt to an environment by observing the decisions made by other people in the network. The transmission of shared information was deliberately constrained by limiting the number of network connections. Each participant could only observe and be observed by their immediate neighbours, but of course the decisions being made by one's immediate neighbours were themselves influenced by broader networks consisting of the individuals those neighbours were interacting with. So, there were both direct and indirect contacts. The researchers linked task-related neural activity with the model-derived cognitive variables important for implementing learning, recording simultaneous brain activity with functional magnetic resonance imaging in a proportion of participants.

Although the experimental paradigm was complex, the outcome of this unique experiment has real-world implications. As the authors state, social networks are thought to play a key part in large-scale social phenomena such as vaccine hesitancy or alleged ‘fake news’. They conclude that there is neural evidence for network-dependent filtering of social information, such that knowledgeable or successful individuals tend to become more highly connected, signalling greater capability or social status to other individuals. We may have evolved to follow ‘influencers’ whom we recognise as better connected than ourselves, for good or ill.

Jiang Y, Mi Q, Zhu L. Neurocomputational mechanism of real-time distributed learning on social networks. Nat Neurosci 2023; 26(3): 506–16.36797365 10.1038/s41593-023-01258-y

